# Outcomes after surgical resection of pulmonary carcinoid tumors

**DOI:** 10.1186/s13019-016-0424-0

**Published:** 2016-03-02

**Authors:** Ikenna C. Okereke, Angela M. Taber, Rogers C. Griffith, Thomas T. Ng

**Affiliations:** Cardiothoracic Surgery, University of Texas Medical Branch, 301 University Blvd, Galveston, TX 77555 USA; Division of Oncology, Warren Alpert Medical School of Brown University, Providence, RI USA; Department of Pathology and Laboratory Medicine, Warren Alpert Medical School of Brown University, Providence, RI USA; Department of Surgery, Warren Alpert Medical School of Brown University, Providence, RI USA

**Keywords:** Carcinoid, Lymphadenopathy, Mitosis

## Abstract

**Background:**

Pulmonary carcinoid tumors make up approximately one percent of all pulmonary tumors, and controversy exists regarding management and prognosis. We undertook a retrospective analysis of all patients who underwent surgical resection of pulmonary carcinoid tumors at our institution.

**Methods:**

From 1992 through 2014, 121 patients who underwent surgical resection of pulmonary carcinoid tumors were retrospectively reviewed. Patient demographics, pathologic data and long-term outcomes were recorded.

**Results:**

There were 96 patients with typical carcinoid tumors and 25 patients with atypical carcinoid tumors. All patients received complete resection of their tumors, with 90 % (109/121) of patients undergoing anatomic resection. There were no peri-operative mortalities. Eighty-one percent (98/121) of patients were female. Mean age was 60.7 years. Five and ten year survival rates were 96 % and 88 % respectively for typical carcinoid tumors, as compared to 87 % and 69 % respectively for atypical carcinoid tumors. Tumor size was not associated with survival (*p* = 0.98). Nodal metastases were evident in 8 % (8/96) of typical carcinoid tumors and 28 % (7/25) percent of atypical carcinoid tumors. Among typical carcinoid cases, the presence of nodal metastases were not associated with overall survival (*p* = 0.55). Among atypical carcinoid cases, the presence of nodal metastases also was not associated with survival (*p* = 0.53). No patients received neoadjuvant or adjuvant chemoradiation treatment.

**Conclusions:**

Excellent long-term outcomes can be achieved following surgical resection of pulmonary carcinoid tumors. The presence of nodal metastases was not associated with overall survival. Tumor size was not associated with either recurrence rates or survival.

## Background

Pulmonary carcinoid tumors make up approximately 1–2 % of all pulmonary tumors [1]. They are derived from the Kulchitsky cell and generally have indolent growth patterns. Carcinoid tumors are categorized as typical or atypical, based on number of mitoses per high power field and presence of necrosis [2]. Surgical resection is the standard treatment for pulmonary carcinoid tumors [3]. There exists debate, however, about the use of adjuvant treatment in patients with locoregional metastases or patients with atypical tumors [4, 5]. We undertook a retrospective review to determine the outcomes of patients with pulmonary carcinoid tumors who underwent surgical resection.

## Methods

After approval by the institutional review board, a database search for pulmonary carcinoid tumors was performed from pathology records of two Brown University hospitals (The Rhode Island Hospital and The Miriam Hospital) over a 22 year period (1992–2014). Over this time span, 121 patients with pulmonary carcinoid tumors underwent surgical resection. Patients who were incidentally found to have a carcinoid tumorlet as part of their resection specimen were not included in the study. Pathologic confirmation of carcinoid tumor was established by a pathologist in all cases. Tumors were classified as typical carcinoid if there were less than 2 mitoses per 2 square millimeters (mm^2^) and no necrosis. Tumors were classified as atypical if there were between 2 and 10 mitoses per mm^2^ or evidence of necrosis. Patient demographics, use of chemotherapy or radiation, peri-operative variables, type of surgical resection, recurrence rates and survival were analyzed retrospectively. Anatomic resection consisted of either segmentectomy, lobectomy, bi-lobectomy or pneumonectomy. Follow-up status was obtained from institutional records and verified by the national social security database.

The Mann–Whitney test was used to compare differences in continuous variables between different tumor groups. Fisher’s exact and Chi-squared tests were used to compare categorical variables. Univariate survival analysis was performed using Kaplan-Meier curves compared with log rank or Cox regression analysis. Multivariate survival analysis was performed with Cox regression analysis, using variables with a p-value of less than 0.1 on univariate analysis.

## Results

### Patients

A list of patient demographics is shown in Table 1. Eighty-one percent (98/121) of the patients were female. Mean age was 60.7 years (11–81). Mean follow-up time was 70.2 months (1–259).

**Table 1:** Demographics

N	121
Age (Mean)	60.7 ± 13.5
Female	81 % (98/121)
Diabetes mellitus	16 % (19/121)
Cardiac disease	12 % (14/121)
Follow-up, months (mean)	70.2 (1–259)

### Surgical management

Anatomic resection was performed in 89 % (108/121) of patients. All patients had complete resection of their tumor. Eleven percent (13/121) of patients underwent resection using a minimally invasive, video-assisted approach. There were no peri-operative mortalities. Mean length of stay was 4.9 days.

### Pathologic analysis

Pathologic results are shown in Table 2. Mean size was 2.3 centimeters (cms). Twenty-one percent (25/121) of patients had an atypical carcinoid tumor. Nodal metastases were present in 8 % (8/96) of patients with typical carcinoid tumors and 28 % (7/25) of patients with atypical carcinoid tumors (*p* < 0.01, Table 3). Patients with atypical carcinoid tumors had greater tumor size than patients with typical carcinoid tumors (2.0 cm vs. 3.2 cm, *p* < .01). Patients with atypical carcinoid tumors were also more likely to have increased number of lymph node metastases than patients with typical carcinoid tumors (*p* < 0.01). In patients with atypical carcinoid tumors, the mean number of mitoses per high power field was 3.9.

**Table 2:** Pathologic results

Typical histology	79 % (96/121)
Anatomic resection	89 % (108/121)
Size, cm (mean)	2.3 ± 1.4
Stage I	85 % (103/121)
II	7 % (8/121)
III	8 % (10/121)

**Table 3:** Typical vs. atypical histology

	Typical (*N* = 96)	Atypical (*N* = 25)	*p*-value
Age, years (mean)	60.0 ± 13.2	63.3 ± 9.8	0.24
Tumor size, cm (mean)	2.0 ± 1.3	3.2 ± 2.2	**<0.01**
Positive lymph node status	8 % (8/96)	28 % (7/25)	**<0.01**
Number of lymph node metastases	0.1	0.6	**<0.01**
Disease-free survival			**0.02**
Overall survival			0.09

Bold values indicate statistically significant results

### Chemoradiation

No patient received neoadjuvant chemotherapy or radiation treatment. Similarly, no patient received adjuvant treatment in the early postoperative period. All patients who experienced recurrent disease received chemotherapy and/or radiation treatment. The usual chemotherapy regimen consisted of cisplatin-based treatment. The decision to use chemotherapy and/or radiation therapy was individualized in each of these patients and based on extent and location of recurrence and presence of symptoms.

### Disease-free survival and overall survival

At the time of last follow-up, 88 % (107/121) of patients were alive in the overall cohort. Five and ten year survival overall were 93 % and 84 % respectively. Of the patients with typical carcinoid tumor, 89 % (85/96) were alive without disease, 1 % (1/96) was alive with distant recurrent disease, and 10 % (10/96) had died without disease. Five and ten year survival in patients with typical carcinoid were 96 % and 88 % respectively.

Of the patients with atypical carcinoid tumor, 76 % (19/25) were alive without disease, 8 % (2/25) were alive with distant recurrent disease, 12 % (3/25) died with disease, and 4 % (1/25) died without disease. Two of the 3 patients who died with disease had distant recurrent disease, while the other patient had both distant and local recurrences. Five and ten year survival in patients with atypical carcinoid were 87 % and 69 % respectively.

**Fig. 1:**
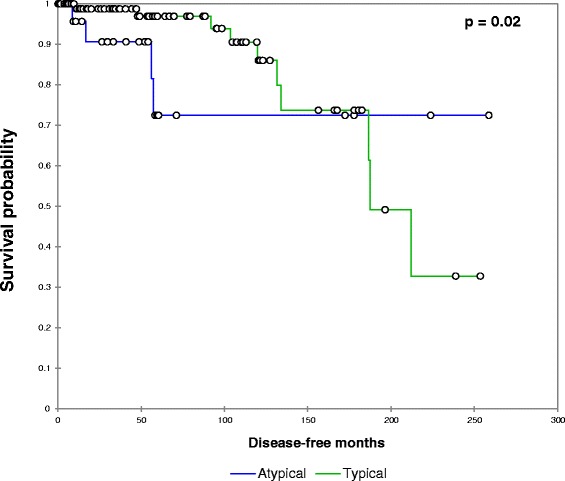
Relationship between typical/atypical histology and disease-free survival. Patients with atypical histology experienced a significantly worsened disease-free survival

Disease-free survival was significantly worsened by increasing age (*p* = 0.03) and atypical histology (*p* = 0.02, Fig. 1). Disease-free survival was not associated with anatomic resection (*p* = 0.91), size (*p* = 0.11), positive lymph node status (*p* = 0.44) or number of lymph node metastases (*p* = 0.13).

Using univariate analysis, factors which were associated with overall survival were age (*p* = 0.03) and number of mitoses (*p* < 0.01). Overall survival was not affected, however, by typical/atypical histology (*p* = 0.09), positive lymph node status (*p* = 0.55) or anatomic resection (*p* = 0.78). When multivariate analysis was used, including all variables with a p value less than .1, only the number of mitoses was significantly associated with survival (*p* = 0.04).

## Discussion

Overall survival for pulmonary carcinoid tumors is excellent, and surgical resection remains the standard of care for these tumors [6]. There exist many different standards and practices for surgical management, however [7]. All of our patients underwent complete resection of their tumor. In our analysis both overall survival and disease-free survival were not affected by undergoing an anatomic resection. But in our study the overwhelming majority of patients had an anatomic resection performed, and conclusions concerning anatomic resection vs. limited resection are difficult. We feel that an anatomic resection should be favored for carcinoid tumors, and limited resections should be considered only for patients who lack sufficient pulmonary reserve.

Similarly, presence or number of lymph node metastases did not affect survival in our study. But given the relatively small number of patients in our study with nodal involvement, caution should be taken in determining the prognostic impact of nodal status. And other studies have shown that nodal involvement does have an impact on outcome of patients with carcinoid tumors [8]. It does seem, however, that patients can live for extended periods of time with microscopic disease, given the indolent nature of carcinoid tumors. Our surgical practice has been to perform a hilar and mediastinal lymph node dissection similar to what we perform for patients with non-small cell lung cancer.

Atypical carcinoid tumors do appear to have more aggressive biology, with statistically increased tumor size and propensity for lymph node metastases [9–12]. And while patients with atypical carcinoid tumors were more likely to develop recurrent disease, overall survival was not affected by typical/atypical histology. This trend occurred because the patients who developed recurrent disease were able to survive for prolonged periods without significant symptomatology or complications [13, 14]. Though it appears that patients with atypical carcinoid tumors are more likely to recur, they can live with disease without considerable morbidity for prolonged periods.

Given the long time frame of the study, preoperative staging and postoperative surveillance strategy varied widely among the cohort [15]. In the early part of this study computed tomography (CT) scan only was used to stage the patient. Now we obtain positron emission tomography – computed tomography (PET-CT) scans during the preoperative workup of our patients. Our current postoperative surveillance strategy has been to perform CT scan evaluation every 6 months for the first 2 years, and then yearly thereafter until year 5. After that, CT scan is performed only if there is suspicion of recurrence based on symptomatology or clinical findings. In our study, only 1 patient recurred after 5 years from resection. That patient is currently alive with disease but no symptoms.

There exist multiple different practice patterns regarding the use of chemotherapy to treat pulmonary carcinoid tumors [16–19]. Chemotherapy is generally not recommended to treat a patient with typical carcinoid tumors who recurs, as typical carcinoid tumors are generally less responsive to chemotherapy [20]. The 1 patient in our study with typical carcinoid who recurred was, as such, treated with radiation therapy only. Patients with atypical carcinoid tumors who recur may experience a survival benefit with chemotherapy.

Along with the retrospective nature of the series, our study is limited by other factors. The overall number of patients in the series is relatively low, but the overall and disease-free survival rates were similar compared to other studies. And because disease recurrences were so infrequent, even in patients with atypical carcinoid tumors, it was difficult to determine a group of patients with a recurrence risk great enough to warrant chemoradiation therapy. A larger multi-center study would potentially identify patients who would benefit from adjuvant treatment, such as a patient with an atypical carcinoid tumor with a very high number of mitoses.

## Conclusions

Patients with pulmonary carcinoid tumors can experience excellent long term outcomes following complete resection of their tumors. Presence of lymph node metastases and tumor size do not appear to affect recurrence rates or survival. Further multi-center studies may potentially identify a high-risk cohort that would benefit from neoadjuvant or adjuvant chemotherapy or radiation treatment.

## References

[1] Naslund A, Rostad H, Strom E, Lund M, Strand T (2011). Carcinoid lung tumors—incidence, treatment and outcomes: a population-based study. Eur J Cardiothorac Surg.

[2] Tsuta K, Raso M, Kalhor N, Liu D, Wistuba I, Moran C (2011). Histologic features of low and intermediate-grade neuroendocrine carcinoma (typical and atypical carcinoid tumors) of the lung. Lung Cancer.

[3] Dahabreh J, Stathopoulos G, Koutantos J, Rigatos S (2009). Lung carcinoid tumor biology: treatment and survival. Oncol Rep.

[4] Hage R, de la Riviere A, Seldenrijk C, Van den Bosch J (2003). Update in pulmonary carcinoid tumors: a review article. Ann Surg Oncol.

[5] Mackley H, Videtic G (2006). Primary carcinoid tumors of the lung: a role for radiotherapy. Oncology.

[6] Kyriss T, Maier S, Veit S, Fritz P, Toomes H, Friedel G (2006). Carcinoid lung tumors: long-term results from 111 resections. GMS Thorac Surg Sci.

[7] Filosso P, Rena O, Donati G, Casadio C, Ruffini E, Papalia E (2002). Bronchial carcinoid tumors: surgical management and long-term outcome. J Thorac Cardiovasc Surg.

[8] Fox M, Van Berkel V, Bousamra M, Sloan S, Martin R (2013). Surgical management of pulmonary carcinoid tumors: sublobar resection versus lobectomy. Amer J Surg.

[9] Thomas C, Tazelaar H, Jett J (2001). Typical and atypical pulmonary carcinoids: outcome in patients presenting with regional lymph node involvement. Chest.

[10] Bini A, Brandolini J, Cassanelli N, Davolr F, Dolci G, Selltri F (2008). Typical and atypical pulmonary carcinoids: our institutional experience. Interact Cardiovasc Thorac Surg.

[11] Garcia-Yuste M, Matilla J (2014). The significance of histology: typical and atypical bronchial carcinoids. Thorac Surg Clin.

[12] Wurtz A, Benhamed L, Conti M, Bouchindhomme B, Porte H (2009). Results of systematic nodal dissection in typical and atypical carcinoid tumors of the lung. J Thorac Oncol.

[13] Canizares M, Matilla J, Cueto A, Algar J, Muguruza I, Moreno-Mata N, Moreno-Balsalobre R (2014). Atypical carcinoid tumours of the lung: prognostic factors and patterns of recurrence. Thorax.

[14] Cardililo G, Sera F, Di Martino M, Graziano P, Giunti R, Carbone L (2004). Bronchial carcinoid tumors: nodal status and long-term survival after resection. Ann Thorac Surg.

[15] Lou F, Sarkaria I, Pietanza C, Travis W, Roh M, Sica G (2013). Recurrence of pulmonary carcinoid tumors after resection: implications for postoperative surveillance. Ann Thorac Surg.

[16] Wirth L, Carter M, Janne P, Johnson B (2004). Outcome of patients with pulmonary carcinoid tumors receiving chemotherapy or chemoradiotherapy. Lung Cancer.

[17] Ohta Y, Toda A, Ohta N, Oda M, Minato H, Nonomura A (2002). An atypical lung carcinoid tumor resected after induction therapy with involvement of the superior sulcus region: report of a case. Surg Today.

[18] Granberg D, Eriksson B, Wilander E, Grimfjard P, Fjallskog M, Oberg K (2001). Experience in treatment of metastatic pulmonary carcinoid tumors. Ann Oncol.

[19] Carretta A, Ceresoli G, Arrigoni G, Canneto B, Reni M, Cigala C (2000). Diagnostic and therapeutic management of neuroendocrine lung tumors: a clinical study of 44 cases. Lung Cancer.

[20] Horsch D, Schmid K, Anlauf M, Darwiche K, Denecke T, Baum R (2014). Neuroendocrine tumors of the bronchopulmonary system (typical and atypical carcinoid tumors): current strategies in diagnosis and treatment. Conclusions of an expert meeting February 2011 in Weimar, Germany. Oncol Res Treat.

